# Macular Changes Observed on Optical Coherence Tomography Angiography in Patients Infected With Human Immunodeficiency Virus Without Infectious Retinopathy

**DOI:** 10.3389/fmed.2022.820370

**Published:** 2022-04-07

**Authors:** Kui-Fang Du, Xiao-Jie Huang, Chao Chen, Wen-Jun Kong, Lian-Yong Xie, Hong-Wei Dong, Wen-Bin Wei

**Affiliations:** ^1^Department of Ophthalmology, Beijing Youan Hospital, Capital Medical University, Beijing, China; ^2^Department of Infectious Diseases, Beijing Youan Hospital, Capital Medical University, Beijing, China; ^3^Beijing Tongren Eye Center, Beijing Key Laboratory of Intraocular Tumor Diagnosis and Treatment, Beijing Ophthalmology and Visual Sciences Key Lab, Beijing Tongren Hospital, Capital Medical University, Beijing, China

**Keywords:** HIV, macular, non-infectious, microvasculopathy, microvasculature, optical coherence tomography angiography, structure

## Abstract

**Purpose:**

As the human immunodeficiency virus (HIV) pandemic is far from over, whether there are subclinical macular changes in HIV-positive patients is something that should not be overlooked. We aimed to apply optical coherence tomography angiography (OCTA) to assess the macular structure and microvasculature changes in patients with HIV without infectious retinopathy.

**Methods:**

HIV-positive and -negative participants were included and classified into three groups: HIV-negative, HIV-positive, and HIV-positive with microvasculopathy. OCTA parameters regarding macular structure and microvasculature were analyzed.

**Results:**

Compared with the HIV-negative group, the superficial retinal vessel density (VD) in the parafovea sectors and the whole Early Treatment of Diabetic Retinopathy Study (ETDRS) grid and the choroidal vascularity index (CVI) in the whole ETDRS grid were significantly decreased in the HIV-positive and HIV-positive with microvasculopathy groups (*p* < 0.05). No differences were found in OCTA parameters between the HIV-positive and HIV-positive with microvasculopathy groups. Retinal, retinal nerve fiber layer-ganglion cell layer-inner plexiform layer (RNFL-GCL-IPL), RNFL, GCL-IPL, and INL thickness showed a negative association with the duration of HIV diagnosis or antiretroviral therapy (ART) (all *p* < 0.05). All OCTA microvasculature parameters showed no association with HIV-related clinical variables (all *p* > 0.05).

**Conclusions:**

Subclinical macular changes existed in HIV-infected patients without clinical infectious retinopathy. Substructures from inner retinal layers might be associated with HIV infection or ART duration.

## Introduction

The human immunodeficiency virus (HIV) pandemic has lasted four decades. Although remarkable achievements have been witnessed in it, it is still far from over (1). Since universal recommendations in the mid-90s, the “treat-all” policy on antiretroviral therapy (ART) greatly contributed toward reducing the visual morbidity and mortality of HIV infection (2–5). However, a higher risk of non-AIDS comorbidities, such as cardiovascular diseases, bone disease, renal and hepatic dysfunction, and neurological disease and cancers should be considered (3, 4).

There were hypotheses about accelerated neuroretinal degeneration because of persistent HIV infection and ART or other factors (6). For patients with HIV infection without ocular opportunistic infections, subtle macular or peripapillary changes were confirmed by various previous studies (7–9). Some results or associations between these structural changes and clinical features were inconsistent (9–11), which might be due to differences in research design and detection machinery.

Optical coherence tomography angiography (OCTA) is a non-invasive instrument possessing an advantage to image retinal and choroidal blood flow and obtain quantitative thickness measurements (12, 13). Besides the wide applications of OCTA in various fundus diseases (13–17) and many systemic diseases (18–20), this non-invasive technology had been proved to be feasible for detecting microvasculopathy in patients with HIV/AIDS with and without clinical retinal diseases (21, 22).

We sought to apply OCTA to assess the macular structure and microvasculature changes in patients with HIV, while there were no clinical infectious retinopathy or only asymptomatic cotton wool spots.

## Materials and Methods

### Participant Recruitment

This prospective cohort study was conducted between August 2019, and October 2019, and approved by the ethics committee of Beijing Youan Hospital, Capital Medical University.

Patients with or without HIV infection were initially included. Patients with a history of diabetes mellitus and cardiovascular and cerebrovascular diseases were excluded. After a routine ocular examination of the anterior segment and fundus, eyes without visible ocular abnormalities or with HIV microvasculopathy were also included. HIV microvasculopathy was diagnosed as asymptomatic cotton wool spots by ophthalmoscopy, which could coexist with small intraretinal hemorrhages, microaneurysms, and telangiectasia (23, 24). The HIV-infected group in this study was defined as having no visible ocular abnormalities in patients with HIV infection. Participants were classified into three groups: HIV-negative, HIV-positive, and HIV-positive with microvasculopathy. Only the right eye was selected. The left eye was recruited when the right eye was substandard. Eyes with a history of intraocular surgery, ocular trauma, amblyopia, glaucoma, and other ocular diseases were excluded. The study was reviewed and approved by the ethics committee of Beijing Youan Hospital, Capital Medical University (LL-2018-150-K). Written informed consent was obtained from all participants.

### Ocular and Systemic Examinations

The main ocular examinations included slit-lamp biomicroscopy for anterior segment, fundus imaging by Optos Daytona, and axial length by Zeiss IOL Master 500.

HIV infection was identified by self-reporting and a previous positive test. For patients with HIV infection, critical HIV-related parameters were collected, including the duration of infection, duration of ART, CD4/CD8, nadir CD4 counts, and blood HIV-RNA.

### Macular Measurements

The OCT device (VG200; SVision Imaging, Ltd., Luoyang, China) used for macular examination was the same as described in previous studies (21, 25). It is a swept-source (SS) OCT with a central wavelength of 1,050 nm (990–1,100 nm full width) and a scanning rate of 200,000 A-scans per second. With automatical pupil focusing and OCT focusing, both 3 × 3 mm and 6 × 6 mm scan patterns centering on the fovea were conducted in this study. Rescan was conducted if the image quality was dissatisfied with apparent flaws. Images with a high signal strength index (SSI) were further evaluated, while images with flow projection artifacts or camera artifacts were excluded.

OCTA parameters were automatically calculated. For segmentation errors, manual corrections can be used to fix them. Macular sectors were performed according to the Early Treatment of Diabetic Retinopathy Study (ETDRS) grid, which was also described in our previous study (21). Within the ETDRS grid, parameters from the inner circle were further classified into the central fovea and parafovea (temporal, superior, nasal, and inferior); while parameters from the outer circle were excluded because of an incomplete edge in the 6 × 6 mm scanning area.

OCTA parameters were stratified into macular microvasculature and macular structural parameters. Microvasculature parameters included the foveal avascular zone (FAZ) from a 3 × 3 mm scan pattern, superficial retinal vessel density (VD), inner retinal VD, and choroidal vascularity index (CVI) from a 6 × 6 mm scan pattern, which also contained quantitative measurements of retinal and choroidal thicknesses. Besides, there were detailed measurements of the retinal nerve fiber layer (RNFL), ganglion cell layer (GCL), inner plexiform layer (IPL), RNFL, GCL-IPL, and INL from the inner retinal layer, as well as photoreceptor-retinal pigment epithelium (PR-RPE). The classification of each OCTA parameter was also described in our previous study (21).

### Statistical Methods

SPSS 25.0 software was used for all statistical analyses. Mann-Whitney test was used for HIV-related variables between HIV-positive and HIV-positive with microvasculopathy groups. Analysis of variance and chi-square test were used for continuous normal distributed and categorical variables, respectively. Differences in macular parameters between groups were tested by one-way ANOVA and Bonferroni tests. Multivariable linear regression analysis was performed between clinical variables and each OCTA parameter in the whole ETDRS grid. *p* < 0.05 was considered statistically significant.

## Results

After excluding participants who did not meet the inclusion criteria, this study included 36 controls without HIV infection (29 males), 46 patients with HIV infection (40 males), and 20 patients with HIV infection with microvasculopathy (19 males). Age, gender, axial length (AL), and signal strength index was not significantly different between the groups (*p* > 0.05). The duration of HIV infection and blood HIV-RNA were not different between the HIV-positive and HIV-positive with microvasculopathy groups (*p* = 0.061 and 0.052, respectively). The ART duration and CD4/CD8 and CD4 levels in the HIV-positive with microvasculopathy group were significantly different from those in the HIV-positive group (*p* = 0.006, 0.001, and *p* < 0.001, respectively) (Table 1).

**Table 1:** Systemic variables and clinical characteristics of participants in this study.

**Characteristic**	**HIV-positive** **(*N* = 46)**	**HIV-positive with microvasculopathy (*N* = 20)**	**HIV-negative (*N* = 36)**	***p*-value**
Age (yrs), mean ± SD	36.2 ± 10.0	36.5 ± 9.1	40.0 ± 9.7	0.181
Gender (no. male: no. female)	40:6	19:1	29:7	0.390
Axial length (mm), median (IQR)	24.0 (23.2, 25.9)	24.3 (23.2, 25.0)	24.0 (23.5, 25.2)	0.961
CD4 count (μl), median (IQR)	102.0 (45.5, 262.5)	16.0 (8.0, 36.0)	/	<0.001*
CD4/CD8, median (IQR)	0.13 (0.07, 0.31)	0.04 (0.03, 0.09)	/	0.001*
HIV-RNA(log), median (IQR)	4.4 (1.3, 5.3)	5.3 (3.4, 5.6)	/	0.052
Duration of HIV infection (mos), median (IQR)	4.0 (0.8, 78.0)	1.0 (0.5, 12.0)	/	0.061
Duration of ART (mos), median (IQR)	1.0 (0, 27.0)	1.0 (0.5, 12.0)	/	0.006*
SSI (3*3), mean ± SD	202.5 ± 121.89	193.6 ± 91.35	229.9 ± 121.26	0.178
SSI (6*6), mean ± SD	187.8 ± 94.22	183.3 ± 83.43	215.4 ± 107.17	0.163

N, number of patients; ART, antiretroviral therapy; HIV, human immunodeficiency virus; SD, standard deviation; SSI, signal strength index; IQR, interquartile range; p-value < 0.05 is indicated by an asterisk (^*^).

**Figure 1:**
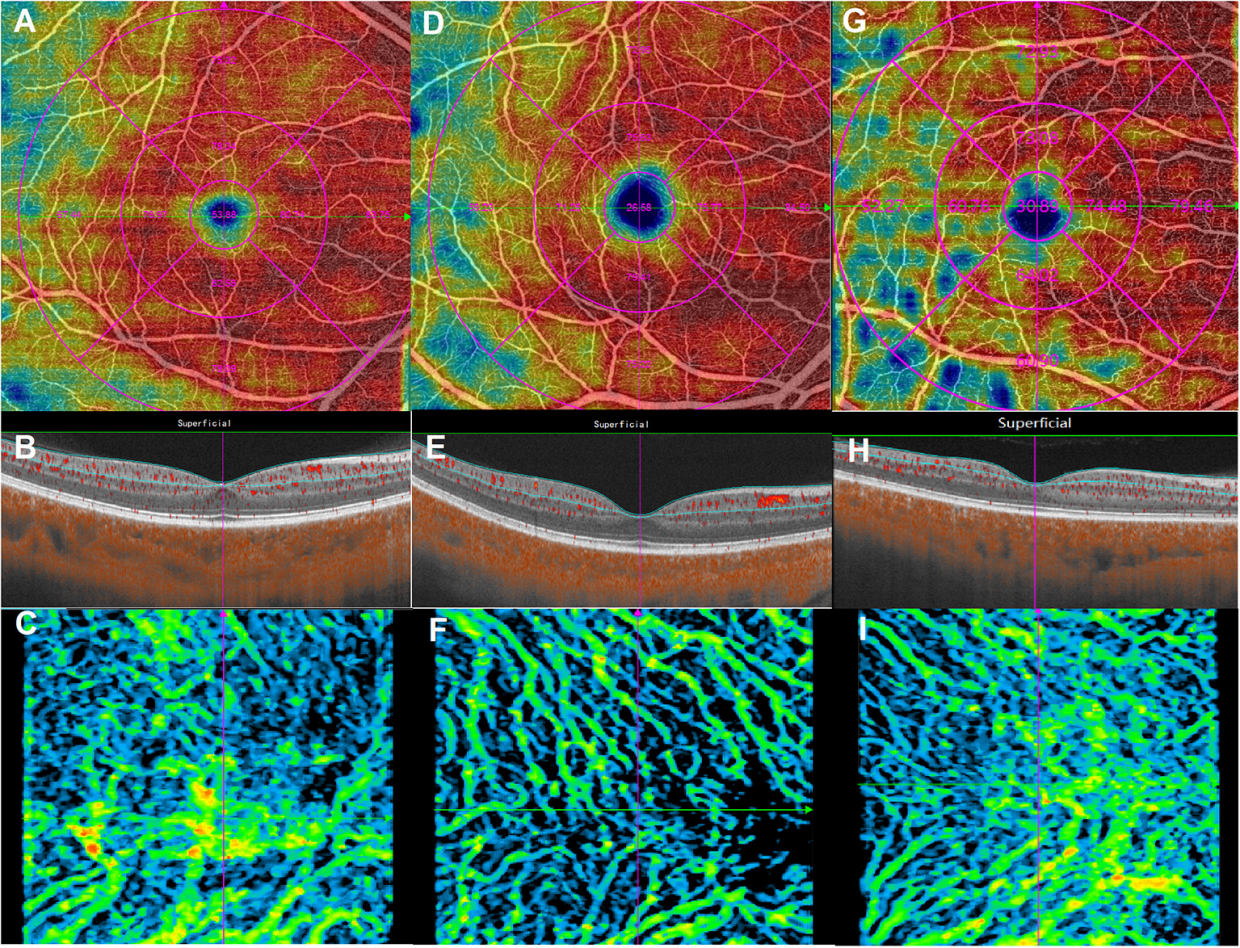
Macular microvasculature images from the 6 × 6 mm scan model: en face optical coherence tomography angiography images of the superficial retinal vessel density (upper), B-scans (middle), and choroidal vascularity index (lower). **(A–C)** are from the same eye in the human immunodeficiency virus (HIV)-negative group; **(D–F)** are from the same eye in the HIV-positive group; **(G–I)** are from the same eye in the HIV-positive with microvasculopathy group.

All OCTA parameters were automatically calculated. All recruited eyes from each group showed no segmentation errors, and no manual correction was used in this study. Microvascular and structural OCTA parameters from the groups are shown in Supplementary Table 1. For macular microvascular variables, compared with the HIV-negative group, superficial retinal VD in all parafovea and the whole ETDRS grid were significantly decreased in the HIV-positive group and HIV-positive with microvasculopathy groups (*p* < 0.05), while CVI in the whole ETDRS grid was also significantly decreased in these two groups (*p* < 0.001) (Table 2; Figure 1).

**Table 2:** Statistical results of macular microvasculatural parameters between groups.

	**Central fovea**		**Superior**		**Inferior**		**Nasal**		**Temporal**		**Whole ETDRS grid**	
	***F***	***p*-value**	***F***	***p*-value**	***F***	***p*-value**	***F***	***p*-value**	***F***	***p*-value**	***F***	***p*-value**
**Superficial retinal VD**												
Groups	1.052	0.353	8.665	<0.001*	12.586	<0.001*	8.690	<0.001*	15.952	<0.001*	9.868	<0.001*
HIV-positive vs. HIV-negative	/	0.559	/	0.001*	/	<0.001*	/	<0.001*	/	<0.001*	/	0.004*
HIV-positive with microvasculopathy vs. HIV-negative	/	0.808	/	0.008*	/	0.001*	/	0.022*	/	<0.001*	/	0.001*
HIV-positive vs. HIV-positive with microvasculopathy	/	1.000	/	1.000	/	1.000	/	1.000	/	1.000	/	1.000
**Inner retinal VD**												
Groups	0.238	0.788	0.780	0.461	4.093	0.020*	0.534	0.588	0.498	0.609	0.258	0.773
HIV-positive vs. HIV-negative	/	1.000	/	0.751	/	0.092	/	1.000	/	1.000	/	1.000
HIV-positive with microvasculopathy vs. HIV-negative	/	1.000	/	1.000	/	0.030*	/	0.946	/	1.000	/	1.000
HIV-positive vs. HIV-positive with microvasculopathy	/	1.000	/	1.000	/	1.000	/	1.000	/	1.000	/	1.000
**CVI**												
Groups	11.195	<0.001*	18.347	<0.001*	19.270	<0.001*	8.702	<0.001*	8.216	<0.001*	15.992	<0.001*
HIV-positive vs. HIV-negative	/	<0.001*	/	<0.001*	/	<0.001*	/	<0.001*	/	<0.001*	/	<0.001*
HIV-positive with microvasculopathy vs. HIV-negative	/	0.1034	/	0.001*	/	<0.001*	/	0.088	/	0.082	/	0.005*
HIV-positive vs. HIV-positive with microvasculopathy	/	0.218	/	0.994	/	1.000	/	0.759	/	0.946	/	0.606
**FAZ**												
Groups	0.251	0.779	/	/	/	/	/	/	/	/	/	/
HIV-positive vs. HIV-negative	/	1.000	/	/	/	/	/	/	/	/	/	/
HIV-positive with microvasculopathy vs. HIV-negative	/	1.000	/	/	/	/	/	/	/	/	/	/
HIV-positive vs. HIV-positive with microvasculopathy	/	1.000	/	/	/	/	/	/	/	/	/	/

HIV, human immunodeficiency virus; ETDRS, early treatment of diabetic retinopathy study; FAZ, foveal avascular zone; F, the statistic of one-way ANOVA; VD, vessel density; CVI, choroidal vascularity index. p-value < 0.05 is indicated by an asterisk (^*^).

For macular structural variables, retinal thickness, choroidal thickness, RNFL-GCL-IPL thickness, RNFL thickness, and GCL-IPL thickness from all macular quadrants showed no intergroup differences (*p* > 0.05). However, the HIV microvasculopathy group showed a significantly higher INL thickness and lower PR-RPE thickness than those of the HIV-negative group (*p* = 0.021 and 0.023, respectively) (Supplementary Table 2).

Duration of HIV diagnosis and ART duration were strongly related variants, which were divided into two different multivariable linear regression models (Tables 3, 4, respectively). For patients with HIV infection, the thickness of retinal, RNFL-GCL-IPL, RNFL, GCL-IPL, and INL showed a strong negative association with the duration of HIV infection or ART duration (all *p* < 0.05). All OCTA microvasculature parameters (FAZ, superficial retinal VD, inner retinal VD, and CVI) showed no association with HIV-related clinical variables, including CD4, CD4/CD8, blood HIV-RNA, duration of HIV, and duration of ART (*p* > 0.05) (Supplementary Table 3).

**Table 3:** Multivariable linear regression analysis between macular structural parameters in the entire Early Treatment of Diabetic Retinopathy Study grid and systemic variables in all patients with human immunodeficiency virus infection (model one).

	**Retinal thickness**				**Choroidal thickness**		**RNFL-GCL-IPL**				**RNFL**				**GCL-IPL**				**INL**		**PR-RPE**	
	**Uni-variable**		**Multi-variable**		**Uni-variable**		**Uni-variable**		**Multi-variable**		**Uni-variable**		**Multi-variable**		**Uni-variable**		**Multi-variable**		**Uni-variable**		**Uni-variable**	
	***B***	***p*-value**	***B***	***p*-value**	***B***	***p*-value**	***B***	***p*-value**	***B***	***p*-value**	***B***	***p*-value**	***B***	***p*-value**	***B***	***p*-value**	***B***	***p*-value**	***B***	***p*-value**	***B***	***p*-value**
Age	−0.484	0.015*	/	/	0.348	0.772	−0.360	0.005*	/	/	−0.067	0.029*	/	/	−0.293	0.006	/	/	−0.023	0.592	−0.093	0.426
CD4	0.002	0.787	/	/	0.001	0.974	−0.004	0.408	/	/	−0.002	0.183	/	/	−0.003	0.487	/	/	−0.001	0.48	0.008	0.066
CD4/CD8	−8.767	0.170	/	/	−10.154	0.782	−8.265	0.050^†^	/	/	−1.534	0.122	/	/	−6.843	0.048*	/	/	−0.958	0.494	0.897	0.795
HIV-RNA (log)	1.276	0.284	/	/	−0.111	0.987	1.113	0.155	/	/	0.267	0.143	/	/	0.892	0.169	/	/	0.296	0.252	−0.180	0.777
Axial length	3.002	0.041*	/	/	−25.266	0.003*	3.462	<0.001*	2.982	0.005*	1.158	<0.001*	1.087	<0.001*	2.354	0.003*	2.049	0.026*	0.514	0.106	−0.943	0.272
Duration of HIV diagnosis	−0.094	0.007*	−0.094	0.007*	0.166	0.425	−0.082	<0.001*	−0.075	0.002*	−0.020	<0.001*	−0.014	0.001*	−0.063	0.001*	−0.057	0.006*	−0.017	0.023*	0.008	0.690
SSI	−0.015	0.495	/	/	−0.135	0.292	−0.020	0.155	/	/	−0.003	0.412	/	/	−0.018	0.116	/	/	−0.004	0.358	0.010	0.448

ART, antiretroviral therapy; B, non-standardized beta; HIV, human immunodeficiency virus; RNFL, retinal nerve fiber layer; GCL, ganglion cell layer; IPL, inner plexiform layer; INL, inner nuclear layer; PR, photoreceptor; RPE, retinal pigment epithelium; SSI, signal strength index. p-value < 0.1 are indicated by asterisk (); p-value < 0.05 are indicated by asterisk (*).

**Table 4:** Multivariable linear regression analysis between macular structural parameters in the entire Early Treatment of Diabetic Retinopathy Study grid and systemic variables in all patients with human immunodeficiency virus infection (model two).

	**Retinal thickness**				**Choroidal thickness**		**RNFL-GCL-IPL**				**RNFL**				**GCL-IPL**				**INL**		**PR-RPE**	
	**Uni-variable**		**Multi-variable**		**Uni-variable**		**Uni-variable**		**Multi-variable**		**Uni-variable**		**Multi-variable**		**Uni-variable**		**Multi-variable**		**Uni-variable**		**Uni-variable**	
	***B***	***p*-value**	***B***	***p*-value**	***B***	***p*-value**	***B***	***p*-value**	***B***	***p*-value**	***B***	***p*-value**	***B***	***p*-value**	***B***	***p*-value**	***B***	***p*-value**	***B***	***p*-value**	***B***	***p*-value**
Age	−0.484	0.015*	/	/	0.348	0.772	−0.360	0.005*	/	/	−0.067	0.029*	/	/	−0.293	0.006*	/	/	−0.023	0.592	−0.093	0.426
CD4	0.002	0.787	/	/	0.001	0.974	−0.004	0.408	/	/	−0.002	0.183	/	/	−0.003	0.487	/	/	−0.001	0.48	0.008	0.066
CD4/CD8	−8.767	0.170	/	/	−10.154	0.782	−8.265	0.050	/	/	−1.534	0.122	/	/	−6.843	0.048*	/	/	−0.958	0.494	0.897	0.795
HIV-RNA (log)	1.276	0.284	/	/	−0.111	0.987	1.113	0.155	/	/	0.267	0.143	/	/	0.892	0.169	/	/	0.296	0.252	−0.180	0.777
Axial length	3.002	0.041*	/	/	−25.266	0.003*	3.462	<0.001*	3.185	0.003*	1.158	<0.001*	1.095	<0.001*	2.354	0.003*	2.207	0.016*	0.514	0.106	−0.943	0.272
Duration of ART	−0.096	0.016*	−0.096	0.016*	0.352	0.132	−0.096	<0.001*	−0.085	0.002*	0.023	<0.001*	−0.017	<0.001*	−0.073	0.001*	−0.065	0.005*	−0.013	0.132	0.016	0.465
SSI	−0.015	0.495	/	/	−0.135	0.292	−0.020	0.155	/	/	−0.003	0.412	/	/	−0.018	0.116	/	/	−0.004	0.358	0.010	0.448

ART, antiretroviral therapy; B, non-standardized beta; HIV, human immunodeficiency virus; RNFL, retinal nerve fiber layer; GCL, ganglion cell layer; IPL, inner plexiform layer; INL, inner nuclear layer; PR, photoreceptor; RPE, retinal pigment epithelium; SSI, signal strength index. p-value < 0.1 are indicated by asterisk (); p-value < 0.05 are indicated by asterisk (*).

## Discussion

Macular damages in retinochoroid structure and microvasculature were identified by OCTA in patients with HIV infection who were free of infectious retinopathy. Both superficial retinal VD and CVI were significantly decreased in the HIV-positive group and HIV-positive with microvasculopathy groups. INL thickness was increased in the HIV microvasculopathy group. Macular substructures from the inner retinal layer, including RNFL-GCL-IPL, RNFL, GCL-IPL, and INL thickness, were associated with the duration of HIV diagnosis or ART.

HIV microvasculopathy manifested as asymptomatic cotton wool spots (CWSs), which could also be called “HIV retinopathy” or “noninfectious retinopathy” (23, 24). These benign lesions were first described by Holland in 1982 (26). The pathophysiology of HIV microvasculopathy is complicated and ambiguous. CWSs in the imaging of OCTA were accompanied by microvasculature changes, including in non-perfused areas in the periphery of the CWSs (27). A degenerative retinal process after CWSs resolution was identified in patients with HIV retinopathy (28). However, none of the macular parameters showed differences between the HIV infection and HIV microvasculopathy groups. First, this could be attributed to the 3-mm diameter inner circle of the ETDRS chart. All OCTA parameters were captured in the fovea and parafoveal area, while CWSs were located in areas rich in RNFL. Second, the mean duration from HIV diagnosis (4 months in the HIV infection group and 1 month in the HIV microvasculopathy group) is very short for significant changes to have occurred. Macular structure and microvasculature changes after CWSs resolution deserve further investigation.

The HIV epidemic is still a challenge in China (29). Ongoing support and care for patients with HIV are still needed in the future, especially for patients beyond viral suppression (30, 31). Various studies had tried to explore retinal vasculature changes in patients with HIV infection with retinal vascular calibers measurements by fundus photographs (32). Studies about retinochoroid microvasculature using OCTA were limited. A previous study found retinal microvasculature changes, including decreased macular VD and perfusion density, in patients with HIV infection (33), similar to our findings. Although OCTA parameters might be different between various OCTA devices and algorithms, the device with a wavelength of 1,050 nm in this study should have its ascendancy over other OCTA devices with shorter wavelengths (34). We also observed decreased retinochoroid microvasculature parameters in the HIV infection group or HIV microvasculopathy group, including superficial retinal VD and CVI. Macular microvasculature parameters showed no association with HIV diagnosis or ART duration, which faithfully reflected that a short duration from HIV diagnosis or ART was not enough to changes microvasculature.

Retinal structural assessments in patients with HIV infection using OCT had been conducted by various studies (35–37). There were hypotheses about the presence of accelerated neuroretinal degenerations in these HIV-positive patients (6), which were in line with the reduced macular structure and microvasculature in our study. The persistence of inflammatory state in patients living with HIV are associated with non-AIDS morbidities, such as cardiovascular diseases and neoplastic diseases (38). Whether these neuroretinal degenerations were associated with chronic inflammation and persistent HIV reservoirs (3) deserves further studies. Besides, despite effective HIV suppression, the comorbidities and aging of patients with HIV infection were still challenges. Increased mortality and comorbidity were associated with risk factors in certain populations, such as smoking, obesity, drug and alcohol abuse, and drug toxicity (39, 40), which could also be the potential risk for these macular changes in this study.

In this study, various substructures from the inner retinal layers showed a marked negative association with the duration of HIV infection or ART, indicating a degeneration tendency from the persistent HIV infection or ART. It provided a valuable clue for further studies about the relationships between retinal vascular changes and systemic vascular diseases in HIV-positive patients. Such presumed changes could help to monitor or predict systemic vascular diseases in these patients.

There are limitations to this study. Although objective macular parameters regarding structure and microvasculature were ample in this study, data about visual function were not involved, such as color vision, contrast sensitivity, multifocal electroretinography, and visual fields. As HIV infection is turning into a type of chronic disease, the presence of many possible systemic confounders could contribute to observed changes. Further trials with longitudinal data and large sample size are warranted.

## Conclusions

With precise structure and microvasculature measurements, OCTA revealed decreased superficial retinal VD and CVI in patients with HIV infection with microvasculopathy or clinically normal fundus. HIV infection or ART duration could influence the inner retinal layer substructures. The application of OCTA was meaningful in the ocular observation of patients with HIV infection.

## Data Availability Statement

The original contributions presented in the study are included in the article/Supplementary Material, further inquiries can be directed to the corresponding author/s.

## Ethics Statement

The studies involving human participants were reviewed and approved by the Ethics Committee of Beijing Youan Hospital, Capital Medical University (LL-2018-150-K). The patients/participants provided their written informed consent to participate in this study.

## Author Contributions

K-FD and W-BW contributed to the conception of the protocol. K-FD and CC obtained the dataset. K-FD, X-JH, L-YX, and W-JK analyzed the data. K-FD and X-JH contributed to writing the first draft of the manuscript and to the revision and editing of the present version of the manuscript. H-WD and W-BW commented on the manuscript and gave final approval of the version to be published. All authors contributed to the article and approved the submitted version.

## Funding

This study was supported by the Scientific Research Project of Beijing Youan Hospital, CCMU, 2018 (YNKTQN20180201), the National Science and Technology Major Project of China during the 13th Five-Year Plan Period (2017ZX10201101), and the Beijing Excellent Talent Plan (2018000021223ZK04).

## Conflict of Interest

The authors declare that the research was conducted in the absence of any commercial or financial relationships that could be construed as a potential conflict of interest.

## Publisher's Note

All claims expressed in this article are solely those of the authors and do not necessarily represent those of their affiliated organizations, or those of the publisher, the editors and the reviewers. Any product that may be evaluated in this article, or claim that may be made by its manufacturer, is not guaranteed or endorsed by the publisher.

## Abbreviations

HIV: Human immunodeficiency virus
OCTA: Optical coherence tomography angiography
VD: Vessel density
ETDRS: Early Treatment of Diabetic Retinopathy Study
CVI: Choroidal vascularity index
INL: Inner nuclear layer
RNFL: Retinal nerve fiber layer
GCL: Ganglion cell layer
IPL: Inner plexiform layer
ART: Antiretroviral therapy
FAZ: Foveal avascular zone
PR-RPE: Photoreceptor-retinal pigment epithelium
AL: Axial length
CWS: Cotton wool spot.
