# Supplementary material for: Macular Changes Observed on Optical Coherence Tomography Angiography in Patients Infected With Human Immunodeficiency Virus Without Infectious Retinopathy
Source: Front Med (Lausanne). 2022 Apr 7;9:820370. doi: 10.3389/fmed.2022.820370 (PMC9021568; doi:10.3389/fmed.2022.820370)
Supplement: Supplementary file 1 [file Table_1.DOCX]

**Supplementary table 1**. Summary of macular microvascular and structural parameters (mean ± Standard Deviation)

|  | HIV-negative | HIV-positive | HIV-positive with microvasculopathy |
| --- | --- | --- | --- |
|  |  |  |  |
| **FAZ** | 0.34 ± 0.11 | 0.33 ± 0.13 | 0.31 ± 0.11 |
| **Superficial retinal VD** |  |  |  |
| Central fovea | 29.67 ± 8.38 | 26.83 ± 10.97 | 26.70 ± 8.11 |
| Superior parafovea | 80.32 ± 4.51 | 76.32 ± 4.80 | 76.37 ± 4.37 |
| Inferior parafovea | 80.48 ± 3.96 | 75.20 ± 5.58 | 75.03 ± 5.83 |
| Nasal parafovea | 78.46 ± 4.51 | 73.81 ± 5.70 | 74.50 ± 5.00 |
| Temporal parafovea | 75.88 ± 4.19 | 70.19 ± 5.31 | 69.92 ± 5.24 |
| Whole ETDRS grid | 68.96 ± 4.24 | 64.47 ± 5.45 | 64.51 ± 4.46 |
| **Inner retinal VD** |  |  |  |
| Central fovea | 34.77 ± 9.84 | 34.60 ± 11.01 | 36.52 ± 11.84 |
| Superior parafovea | 85.30 ± 3.14 | 84.58 ± 2.67 | 84.56 ± 2.54 |
| Inferior parafovea | 84.95 ± 2.71 | 83.53 ± 2.84 | 82.82 ± 3.33 |
| Nasal parafovea | 83.13 ± 2.87 | 82.47 ± 3.15 | 82.60 ± 2.55 |
| Temporal parafovea | 81.57 ± 3.39 | 81.11 ± 2.88 | 80.79 ± 2.29 |
| Whole ETDRS grid | 73.85 ± 3.60 | 73.26 ± 3.79 | 73.46 ± 3.68 |
| **CVI** |  |  |  |
| Central fovea | 0.48 ± 0.07 | 0.37 ± 0.12 | 0.42 ± 0.10 |
| Superior parafovea | 0.48 ± 0.07 | 0.34 ± 0.12 | 0.37 ± 0.13 |
| Inferior parafovea | 0.49 ± 0.07 | 0.35 ± 0.12 | 0.37 ± 0.11 |
| Nasal parafovea | 0.48 ± 0.09 | 0.36 ± 0.13 | 0.40 ± 0.14 |
| Temporal parafovea | 0.46 ± 0.09 | 0.37 ± 0.11 | 0.39 ± 0.12 |
| Whole ETDRS grid | 0.48 ± 0.06 | 0.36 ± 0.11 | 0.39 ± 0.11 |
| **Retinal thickness** |  |  |  |
| Central fovea | 262.69 ± 15.77 | 260.83 ± 21.90 | 260.35 ± 13.21 |
| Superior parafovea | 345.78 ± 15.14 | 343.33 ± 17.00 | 342.35 ± 13.48 |
| Inferior parafovea | 340.94 ± 16.00 | 337.37 ± 18.79 | 336.00 ± 16.45 |
| Nasal parafovea | 344.17 ± 14.22 | 343.15 ± 18.82 | 340.15 ± 15.70 |
| Temporal parafovea | 330.86 ± 14.45 | 327.17 ± 16.55 | 324.75 ± 14.88 |
| Whole ETDRS grid | 324.89 ± 13.34 | 322..37 ± 16.69 | 320.72 ± 13.60 |
| **Choroidal thickness** |  |  |  |
| Central fovea | 366.4 ± 101.45 | 334.76 ± 97.12 | 354.45 ± 96.38 |
| Superior parafovea | 373.77 ± 99.73 | 349.13 ± 94.59 | 353.45 ± 95.34 |
| Inferior parafovea | 365.97 ± 104.08 | 328.98 ± 99.70 | 350.50 ± 92.55 |
| Nasal parafovea | 334.43 ± 102.25 | 306.87 ± 95.81 | 325.00 ± 92.70 |
| Temporal parafovea | 367.74 ± 96.08 | 344.63 ± 91.12 | 354.45 ± 92.49 |
| Whole ETDRS grid | 361.66 ± 98.42 | 332.87 ± 93.55 | 347.57 ± 91.90 |
| **RNFL-GCL-IPL** |  |  |  |
| Central fovea | 43.58 ± 9.49 | 43.35 ± 11.35 | 45.53 ± 12.15 |
| Superior parafovea | 126.08 ± 8.55 | 124.39 ± 12.34 | 125.21 ± 7.40 |
| Inferior parafovea | 125.19 ± 9.00 | 122.76 ± 13.74 | 123.95 ± 8.24 |
| Nasal parafovea | 120.17 ± 8.79 | 118.78 ± 13.52 | 120.16 ± 8.36 |
| Temporal parafovea | 112.19 ± 8.03 | 109.89 ± 10.64 | 110.89 ± 8.31 |
| Whole ETDRS grid | 105.44 ± 7.61 | 103.84 ± 11.26 | 105.15 ± 7.35 |
| **RNFL** |  |  |  |
| Central fovea | 14.28 ± 0.97 | 14.33 ± 0.99 | 14.95 ± 1.08 |
| Superior parafovea | 31.33 ± 2.77 | 31.76 ± 4.33 | 32.47 ± 2.63 |
| Inferior parafovea | 30.72 ± 3.49 | 31.26 ± 4.45 | 32.32 ± 2.77 |
| Nasal parafovea | 25.78 ± 2.54 | 26.30 ± 3.61 | 26.89 ± 2.47 |
| Temporal parafovea | 21.00 ± 2.18 | 20.39 ± 1.69 | 21.42 ± 1.95 |
| Whole ETDRS grid | 24.62 ± 2.06 | 24.1 ± 2.62 | 25.61 ± 1.75 |
| **GCL-IPL** |  |  |  |
| Central fovea | 29.17 ± 9.13 | 28.85 ± 11.14 | 30.58 ± 11.51 |
| Superior parafovea | 94.75 ± 7.39 | 92.48 ± 9.18 | 93.37 ± 6.95 |
| Inferior parafovea | 94.42 ± 6.79 | 91.48 ± 10.31 | 92.21 ± 6.89 |
| Nasal parafovea | 94.31 ± 7.20 | 92.41 ± 10.75 | 93.21 ± 7.32 |
| Temporal parafovea | 91.17 ± 7.30 | 89.46 ± 9.86 | 89.37 ± 7.43 |
| Whole ETDRS grid | 80.76 ± 6.34 | 78.93 ± 9.18 | 79.75 ± 6.63 |
| **INL** |  |  |  |
| Central fovea | 22.43 ± 3.80 | 23.41 ± 4.77 | 24.32 ± 4.45 |
| Superior parafovea | 45.20 ± 3.79 | 46.78 ± 4.29 | 48.42 ± 4.41 |
| Inferior parafovea | 44.63 ± 4.15 | 45.67 ± 3.68 | 47.42 ± 3.69 |
| Nasal parafovea | 44.83 ± 4.71 | 46.20 ± 4.24 | 46.89 ± 4.46 |
| Temporal parafovea | 42.71 ± 3.64 | 44.72 ± 3.55 | 46.05 ± 3.97 |
| Whole ETDRS grid | 39.96 ± 3.50 | 41.36 ± 3.24 | 42.62 ± 3.58 |
| **PR-RPE** |  |  |  |
| Central fovea | 196.67 ± 8.01 | 193.43 ± 9.70 | 189.26 ± 11.29 |
| Superior parafovea | 174.53 ± 8.94 | 172.24 ± 8.38 | 168.32 ± 10.31 |
| Inferior parafovea | 171.03 ± 9.09 | 169.26 ± 9.76 | 165.00 ± 12.13 |
| Nasal parafovea | 179.00 ± 10.19 | 178.15 ± 9.53 | 174.21 ± 11.29 |
| Temporal parafovea | 178.22 ± 20.20 | 172.98 ± 8.16 | 167.95 ± 11.15 |
| Whole ETDRS grid | 179.89 ± 9.20 | 177.21 ± 8.10 | 172.95 ± 10.54 |

HIV, human immunodeficiency virus; ETDRS, early treatment of diabetic retinopathy study; FAZ, foveal avascular zone; VD, vessel density; CVI, choroidal vascularity index; RNFL, retinal nerve fiber layer; GCL, ganglion cell layer; IPL: inner plexiform layer; INL, inner nuclear layer; PR, photoreceptor; RPE, retinal pigment epithelium.
