# Supplementary material for: Macular Changes Observed on Optical Coherence Tomography Angiography in Patients Infected With Human Immunodeficiency Virus Without Infectious Retinopathy
Source: Front Med (Lausanne). 2022 Apr 7;9:820370. doi: 10.3389/fmed.2022.820370 (PMC9021568; doi:10.3389/fmed.2022.820370)
Supplement: Supplementary file 2 [file Table_2.DOCX]

**Supplementary table 2.** Summary of the statistical results of macular structural parameters between groups

| **Retinal thickness** | Central Fovea | | Superior | | Inferior | | Nasal | | Temporal | | Whole ETDRS grid | |
| --- | --- | --- | --- | --- | --- | --- | --- | --- | --- | --- | --- | --- |
|  | F | p-value | F | p-value | F | p-value | F | p-value | F | p-value | F | p-value |
| Groups | 0.134 | 0.874 | 0.383 | 0.683 | 0.876 | 0.420 | 0.379 | 0.686 | 1.012 | 0.367 | 0.556 | 0.575 |
| HIV-positive vs HIV-negative | / | 1.000 | / | 1.000 | / | 0.569 | / | 1.000 | / | 0.576 | / | 1.000 |
| HIV-positive with microvasculopathy vs HIV-negative | / | 1.000 | / | 1.000 | / | 1.000 | / | 1.000 | / | 0.852 | / | 0.964 |
| HIV-positive vs HIV-positive with microvasculopathy | / | 1.000 | / | 1.000 | / | 1.000 | / | 1.000 | / | 1.000 | / | 1.000 |
| **Choroidal thickness** | Central Fovea | | Superior | | Inferior | | Nasal | | Temporal | | Whole ETDRS grid | |
|  | F | p-value | F | p-value | F | p-value | F | p-value | F | p-value | F | p-value |
| Groups | 1.055 | 0.352 | 0.682 | 0.508 | 1.387 | 0.255 | 0.827 | 0.441 | 0.612 | 0.544 | 0.918 | 0.403 |
| HIV-positive vs HIV-negative | / | 0.466 | / | 0.774 | / | 0.306 | / | 0.632 | / | 0.814 | / | 0.539 |
| HIV-positive with microvasculopathy vs HIV-negative | / | 1.000 | / | 1.000 | / | 1.000 | / | 1.000 | / | 1.000 | / | 1.000 |
| HIV-positive vs HIV-positive with microvasculopathy | / | 1.000 | / | 1.000 | / | 1.000 | / | 1.000 | / | 1.000 | / | 1.000 |
| **RNFL-GCL-IPL** | Central Fovea | | Superior | | Inferior | | Nasal | | Temporal | | Whole ETDRS grid | |
|  | F | p-value | F | p-value | F | p-value | F | p-value | F | p-value | F | p-value |
| Groups | 0.285 | 0.753 | 0.273 | 0.762 | 0.468 | 0.628 | 0.192 | 0.825 | 0.611 | 0.545 | 0.326 | 0.722 |
| HIV-positive vs HIV-negative | / | 1.000 | / | 1.000 | / | 1.000 | / | 1.000 | / | 0.815 | / | 1.000 |
| HIV-positive with microvasculopathy vs HIV-negative | / | 1.000 | / | 1.000 | / | 1.000 | / | 1.000 | / | 1.000 | / | 1.000 |
| HIV-positive vs HIV-positive with microvasculopathy | / | 1.000 | / | 1.000 | / | 1.000 | / | 1.000 | / | 1.000 | / | 1.000 |
| **RNFL** | Central Fovea | | Superior | | Inferior | | Nasal | | Temporal | | Whole ETDRS grid | |
|  | F | p-value | F | p-value | F | p-value | F | p-value | F | p-value | F | p-value |
| Groups | 3.198 | 0.045*^*^* | 0.642 | 0.529 | 1.061 | 0.350 | 0.849 | 0.431 | 2.217 | 0.114 | 1.228 | 0.297 |
| HIV-positive vs HIV-negative | / | 1.000 | / | 1.000 | / | 1.000 | / | 1.000 | / | 0.476 | / | 1.000 |
| HIV-positive with microvasculopathy vs HIV-negative | / | 0.061 | / | 0.781 | / | 0.445 | / | 0.608 | / | 1.000 | / | 0.388 |
| HIV-positive vs HIV-positive with microvasculopathy | / | 0.075 | / | 1.000 | / | 0.955 | / | 1.000 | / | 0.158 | / | 0.600 |
| **GCL-IPL** | Central Fovea | | Superior | | Inferior | | Nasal | | Temporal | | Whole ETDRS grid | |
|  | F | p-value | F | p-value | F | p-value | F | p-value | F | p-value | F | p-value |
| Groups | 0.185 | 0.831 | 0.779 | 0.462 | 1.210 | 0.303 | 0.444 | 0.643 | 0.474 | 0.624 | 0.551 | 0.578 |
| HIV-positive vs HIV-negative | / | 1.000 | / | 0.646 | / | 0.384 | / | 1.000 | / | 1.000 | / | 0.889 |
| HIV-positive with microvasculopathy vs HIV-negative | / | 1.000 | / | 1.000 | / | 1.000 | / | 1.000 | / | 1.000 | / | 1.000 |
| HIV-positive vs HIV-positive with microvasculopathy | / | 1.000 | / | 1.000 | / | 1.000 | / | 1.000 | / | 1.000 | / | 1.000 |
| **INL** | Central Fovea | | Superior | | Inferior | | Nasal | | Temporal | | Whole ETDRS grid | |
|  | F | p-value | F | p-value | F | p-value | F | p-value | F | p-value | F | p-value |
| Groups | 1.203 | 0.305 | 3.861 | 0.024*^*^* | 3.236 | 0.044*^*^* | 1.585 | 0.210 | 5.759 | 0.004*^*^* | 4.013 | 0.021*^*^* |
| HIV-positive vs HIV-negative | / | 0.961 | / | 0.276 | / | 0.689 | / | 0.522 | / | 0.050 | / | 0.209 |
| HIV-positive with microvasculopathy vs HIV-negative | / | 0.405 | / | 0.023*^*^* | / | 0.038 *^*^* | / | 0.319 | / | 0.006*^*^* | / | 0.021*^*^* |
| HIV-positive vs HIV-positive with microvasculopathy | / | 1.000 | / | 0.452 | / | 0.299 | / | 1.000 | / | 0.552 | / | 0.525 |
| **PR-RPE** | Central Fovea | | Superior | | Inferior | | Nasal | | Temporal | | Whole ETDRS grid | |
|  | F | p-value | F | p-value | F | p-value | F | p-value | F | p-value | F | p-value |
| Groups | 3.872 | 0.024*^*^* | 2.991 | 0.055 | 2.266 | 0.109 | 1.475 | 0.234 | 3.478 | 0.035*^*^* | 3.722 | 0.028*^*^* |
| HIV-positive vs HIV-negative | / | 0.384 | / | 0.762 | / | 1.000 | / | 1.000 | / | 0.294 | / | 0.552 |
| HIV-positive with microvasculopathy vs HIV-negative | / | 0.021*^*^* | / | 0.049 | / | 0.109 | / | 0.294 | / | 0.035 *^*^* | / | 0.023*^*^* |
| HIV-positive vs HIV-positive with microvasculopathy | / | 0.327 | / | 0.335 | / | 0.366 | / | 0.468 | / | 0.582 | / | 0.255 |

HIV, human immunodeficiency virus; ETDRS, early treatment of diabetic retinopathy study; RNFL, retinal nerve fiber layer; F, statistic of one-way ANOVA; GCL, ganglion cell layer; IPL, inner plexiform layer; INL, inner nuclear layer; PR, photoreceptor; RPE, retinal pigment epithelium. p-value < 0.05 are indicated by asterisk (^*^).
