# Supplementary material for: Macular Changes Observed on Optical Coherence Tomography Angiography in Patients Infected With Human Immunodeficiency Virus Without Infectious Retinopathy
Source: Front Med (Lausanne). 2022 Apr 7;9:820370. doi: 10.3389/fmed.2022.820370 (PMC9021568; doi:10.3389/fmed.2022.820370)
Supplement: Supplementary file 3 [file Table_3.docx]

**Supplementary table 3.** Multivariable linear regression analysis between macular microvasculature parameters in the whole Early Treatment of Diabetic Retinopathy Study grid and systemic variables in all patients with human immunodeficiency virus infection

|  | **FAZ** | | **Whole Superficial Retinal VD** | | | | **Whole Inner Retinal VD** | | **Whole CVI** | | | |
| --- | --- | --- | --- | --- | --- | --- | --- | --- | --- | --- | --- | --- |
|  | Uni-variable | | Uni-variable | | Uni-variable | | Uni-variable | | Uni-variable | | multi-variable | |
|  | B | p-value | B | p-value | B | p-value | B | p-value | B | p-value | B | p-value |
| Age | 0.001 | 0.571 | -0.045 | 0.495 | / | / | -0.045 | 0.355 | 0.002 | 0.033^*^ | / | / |
| CD4 | 0.000 | 0.936 | -0.002 | 0.490 | / | / | 0.001 | 0.774 | 0.000 | 0.161 | / | / |
| CD4/CD8 | 0.019 | 0.727 | -1.985 | 0.361 | / | / | -1.100 | 0.491 | -0.060 | 0.164 | / | / |
| HIV-RNA(log) | -0.010 | 0.290 | 0.474 | 0.232 | / | / | 0.340 | 0.244 | 0.009 | 0.270 | / | / |
| Axial length | 0.042 | ＜0.001*^*^* | 1.043 | 0.029*^**^* | 1.652 | 0.001*^*^* | 1.099 | 0.001*^*^* | -0.010 | 0.231 | / | / |
| Duration of HIV infection | 0.000 | 0.473 | -0.016 | 0.181 | / | / | -0.009 | 0.287 | 0.000 | 0.860 | / | / |
| Duration of ART | 0.000 | 0.445 | -0.023 | 0.092 | / | / | -0.007 | 0.459 | 0.000 | 0.393 | / | / |
| SSI | 0.000 | 0.439 | 0.014 | 0.053*^**^* | 0.021 | 0.003*^*^* | -0.003 | 0.581 | 0.000 | 0.001*^*^* | 0.000 | 0.001*^*^* |

ART, antiretroviral therapy; B, non-standardized beta; HIV, human immunodeficiency virus; FAZ, foveal avascular zone; VD, vessel density; CVI, choroidal vascularity index; SSI, signal strength index. p-value < 0.05 are indicated by asterisk (^*^).
